# Microbial imbalance in Chinese children with diarrhea or constipation

**DOI:** 10.1038/s41598-024-60683-6

**Published:** 2024-06-12

**Authors:** Jing Ren, Yi Ren, Yu Mu, Lanying Zhang, Binghan Chen, Sisi Li, Qinyi Fang, Zhiming Zhang, Kejian Zhang, Sabrina Li, Wei Liu, Yutao Cui, Xu Li

**Affiliations:** 1 Coyote Bioscience (Beijing) Co., Ltd., Beijing, China; 2 Dr. Cuiyutao Healthcare Co., Ltd., Beijing, China; 3 Coyote Diagnostics Lab (Beijing) Co., Ltd., Beijing, China; 4 State Key Laboratory of Pathogen and Biosecurity, Beijing Institute of Microbiology and Epidemiology, Beijing, China

**Keywords:** Gut microbiota, 16S rRNA sequencing, Childhood diarrhea, Childhood constipation, Cross-sectional study, Microbiology, Biomarkers, Gastroenterology, Medical research

## Abstract

Diarrhea and constipation are common health concerns in children. Numerous studies have identified strong association between gut microbiota and digestive-related diseases. But little is known about the gut microbiota that simultaneously affects both diarrhea and constipation or their potential regulatory mechanisms. Stool samples from 618 children (66 diarrhea, 138 constipation, 414 healthy controls) aged 0–3 years were collected to investigate gut microbiota changes using 16S rRNA sequencing. Compared with healthy, children with diarrhea exhibited a significant decrease in microbial diversity, while those with constipation showed a marked increase (p < 0.05). Significantly, our results firstly *Ruminococcus* increased in constipation (p = 0.03) and decreased in diarrhea (p < 0.01) compared to healthy controls. Pathway analysis revealed that *Ruminococcus* highly involved in the regulation of five common pathways (membrane transport, nervous system, energy metabolism, signal transduction and endocrine system pathways) between diarrhea and constipation, suggesting a potential shared regulatory mechanism. Our finding firstly reveals one core microorganisms that may affect the steady balance of the gut in children with diarrhea or constipation, providing an important reference for potential diagnosis and treatment of constipation and diarrhea.

## Introduction

Pediatric diarrhea and constipation are common diseases in children with serious consequences worldwide^1,2^. Children with diarrhea experience a range of health impacts, such as difficulty in feeding, reduced immunity, malnutrition, and foodborne illness, with potential complications like viral myocarditis, while children with constipation are at risk of various health issues, including mental, behavioral, social, and physical challenges. Pediatric diarrhea, for instance, the second leading cause of death among children under 5 years old, accounts for over 0.5 million child fatalities and affects up to 5% of adults^3^. Pathogens such as rotaviruses, noroviruses, astroviruses^4^, Shigella^5^, pathogenic Y. enterocolitica, Enteropathogenic *Escherichia coli* (EPEC), Cryptosporidium^6^, and others are identified as etiological factors contributing to pediatric diarrhea by inducing intestinal inflammation and damage through invasion. Furthermore, disruptions in gut microbiota equilibrium caused by factors like weaning practices, deficiencies in essential micronutrients like zinc and selenium, and imbalances in probiotic levels can also contribute to the occurrence of diarrhea in children. Growing evidence implicates that moderate to severe diarrhea can lead to decreased bacterial diversity^7^ and altered microbiota composition in children, while the intestinal microbiota in patients with constipation is also disturbed^8^. Critically, intestinal microbial perturbation during infancy is not only associated with adverse outcome, such as diarrhea and necrotizing enterocolitis, but also long-term medical effects^9^: obesity, inflammatory bowel disease, stunting, cognitive impairment are some example. Therefore, the gastrointestinal tract microbiota in early life is key for the development and maturation of the infant mucosal and systemic immune system.

Previous studies have mainly focused on characterizing the overall changes in the diversity and richness of the intestinal microbial community^10^, without specifically investigating the differences and commonalities in the composition and metabolic pathways of the gut microbiota between diarrhea and constipation. It remains unclear whether the underlying pathogenesis of these two conditions is related to dysbiosis of the intestinal microbiota. Understanding this relationship might reveal a potential mutual microbial regulatory mechanism and contribute to the diagnosis and treatment of diarrhea and constipation.

In this study, we utilized 16S rRNA sequencing technology to explore the composition of the intestinal microbiota in children with diarrhea or constipation. For the first time, we have identified a bacterial genera that exhibit reversed relative abundance levels in childhood diarrhea and constipation, and a possible regulatory mechanism for both diseases. These findings provide valuable insights into the common microbial mechanisms underlying the pathogenesis of diarrhea and constipation in children, and establish the foundation for developing microbiome-based interventions to prevent these disorders.

## Results

### Characteristics of the study population

We conducted a comparison of gut microbiome diversity in a discovery cohort comprising healthy children (HC, n = 414), children with constipation (CC, n = 138), and children with diarrhea (CD, n = 66) (Table 1). Fecal samples from these groups were analyzed using high-throughput sequencing of the 16S rRNA gene. The median age of CC group was 1.27 years (interquartile range [IQR]: 0.88, 1.85) with 42.0% children being male, while the median age of CD group was 0.87 years (IQR: 0.55, 1.15) with 59.1% males. The HC group had a mean age of 1.08 years (IQR: 0.65, 1.70) with 54.6% males. Age differences (Kruskal-Walli’s test, p < 0.001) and gender differences (χ^2^ = 11.24, p = 0.02) were observed among the three groups in the discovery cohort. To ensure data consistency between absolute and relative quantification of bacteria, we performed quantitative polymerase chain reaction (qPCR) in a validation cohort consisting of 294 healthy children (H′C′), 146 children with constipation (C′C′), and 47 children with diarrhea (C′D′). The median age of C′C′ group was 1.24 years (IQR: 0.89, 1.81) and 45.9% were male, while the C′D′ group was 0.83 years (IQR: 0.59, 1.24) with 59.6% males. The H′C′ group had a mean age of 1.01 years (IQR: 0.66, 1.69) with 56.1% males. There were age differences (Kruskal–Wallis’s test, p < 0.001) among the three groups in the validation cohort, but no gender differences (χ^2^ = 4.90, p = 0.09).

**Table 1:** The characteristics of the study population.

Characteristics	Constipation	Diarrhea	Healthy_Control	p value
Characteristics of the discovery cohort for 16S rRNA sequencing				
Age (years)				
Median (IQR*)	1.27 (0.88–1.85)	0.87 (0.55–1.15)	1.08 (0.65–1.70)	< 0.001
Age Group (years)				
0–0.5	7 (5.1%)	10 (15.2%)	63 (15.2%)	0.002
0.5–1	41 (29.7%)	31 (47.0%)	131 (31.6%)	
1–2	63 (45.7%)	21 (31.8%)	151 (36.5%)	
2–3	27 (19.6%)	4 (6.1%)	69 (16.7%)	
Gender				
Male	58 (42.0%)	39 (58.5%)	226 (54.6%)	0.020
Female	80 (58.0%)	27 (41.5%)	187 (45.2%)	
Not available	0	0	1 (0.2%)	
Characteristics of the validation cohort for qPCR*				
Age (years)				
Median (IQR)	1.24(0.89–1.81)	0.83(0.59–1.24)	1.01(0.66–1.69)	< 0.001
Age Group				
0–0.5	6 (4.1%)	9 (19.1%)	49 (16.7%)	< 0.001
0.5–1	48 (32.9%)	21 (44.7%)	97 (33.0%)	
1–2	68 (46.6%)	16 (34.0%)	96 (32.7%)	
2–3	24 (16.4%)	1 (2.1%)	52 (17.7%)	
Gender				
Male	67 (45.9%)	28 (59.6%)	165 (56.1%)	0.090
Female	79 (54.1%)	19 (40.4%)	129 (43.9%)	

IQR, interquartile range; qPCR, quantitative polymerase chain reaction.

### Diarrhea associated with a decrease in the diversity of the intestinal microbiota in children while constipation related to an increase

**Figure 1:**
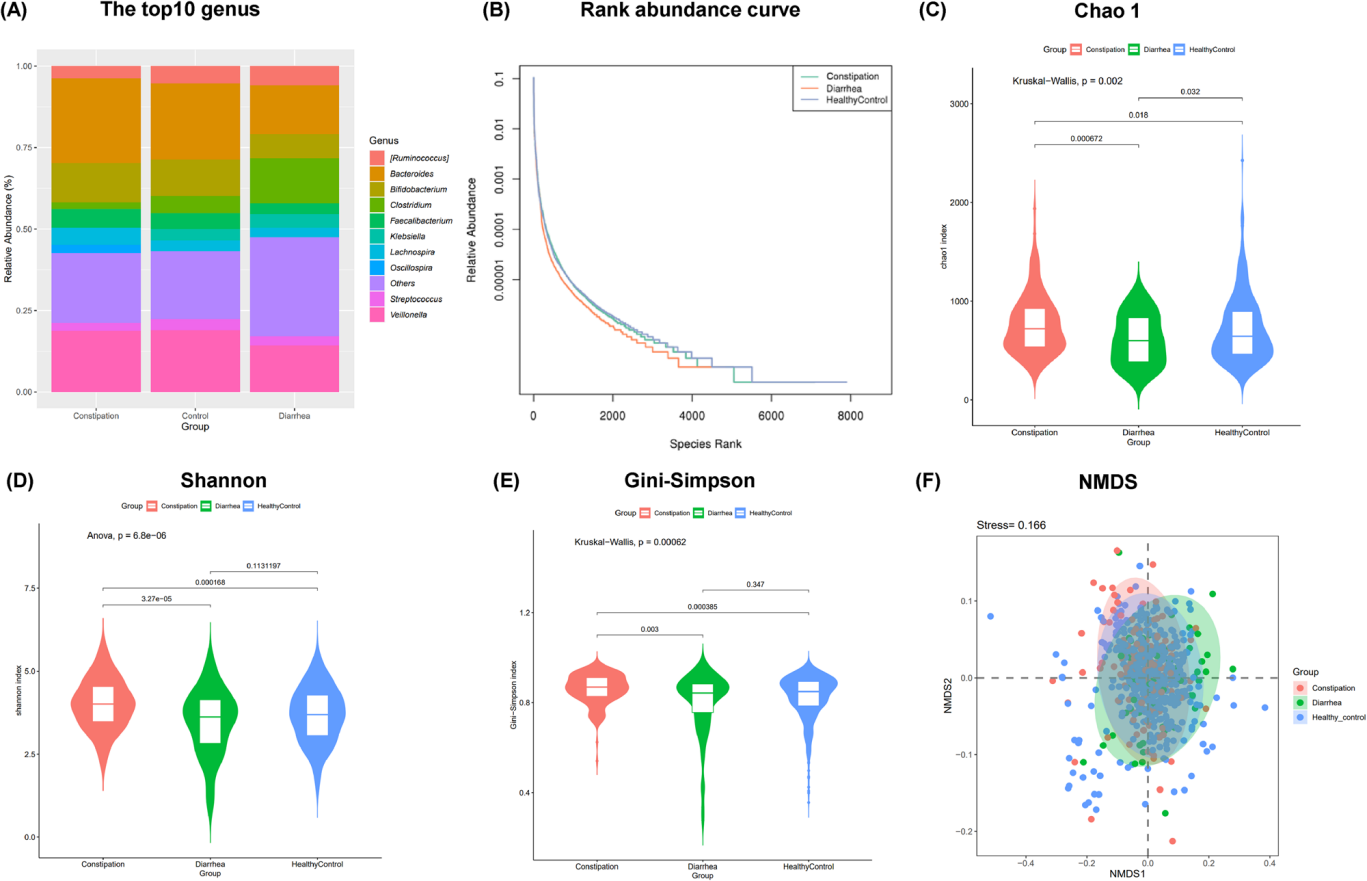
Comparison of gut microbial structure among the CC, CD and HC groups. (**A**) The structure of the gut microbiota at the genus level among the three groups. (**B**) Rank abundance curve showing the relative species abundance and evenness. (**C**) The Chao1 index of the CC group was significantly higher as compared with the HC group (p = 0.018), while that of CD group was significantly lower (p = 0.032). (**D**) The Shannon index of the CC group was significantly higher than that of HC (p < 0.001), while the Shannon index of CD group was lower although the difference was not significant (p = 0.113). (**E**) The Gini-Simpson index of the CC group was significantly higher than that of the HC (p < 0.001), while the Gini-Simpson index in the CD group was lower although the difference was not significant (p = 0.374). (**F**) Beta-diversity index (plots of NMDS) among the three groups.

The operational taxonomic units (OUTs) table of raw counts was normalized to an OTU table of relative abundance values (Supplemental Table S1). Same types of taxa were agglomerated at the phylum, class, order, family, and genus level. At the genus level, 10 most abundant genus, including *Bacteroides*, *Veillonella*, *Bifidobacterium*, Clostridiaceae *Clostridium*, Lachnospiraceae *Ruminococcus*, *Faecalibacterium, Klebsiella*, *Streptococcus*, *Lachnospira* and others, were detected in CD, CC and HC groups (Fig. 1A). The rank-abundance curve (Fig. 1B) showed the relative species abundance and evenness among groups. The α-diversity indexes define the richness and evenness within a microbial community either qualitatively (Chao1 indexes) or quantitatively (Shannon and Simpson indexes). Based on the analysis of alpha diversity, the richness and evenness of microbial community in CC group was significantly increased [Chao1 (p = 0.018, Fig. 1C), Shannon (p < 0.001, Fig. 1D), and Gini-Simpson (p < 0.001, Fig. 1E)] when compared with the HC group, while the richness of the intestinal microbiota in CD group was significantly reduced [Chao1 (p = 0.032, Fig. 1C)]. These results were consistent with prior research^11,12^. After subsequent correction for the effects of age and gender using MaAsLin2, a multivariate analysis by linear models, the richness and evenness of microbial community was still significantly increased in CC groups [Shannon (p < 0.001), and Gini-Simpson (p < 0.001)], and reduced in CD groups [Chao1 (p = 0.040), Shannon (p = 0.023), and Gini-Simpson (p = 0.016)]. To assess the differences between disease groups (CC, CD group) and the healthy group, the beta diversity was evaluated based on the presence or absence of OTUs. The NMDS (Non-Metric Multi-Dimensional Scaling) analysis based on the unweighted Unifrac distances of microbial communities in the samples was shown in Fig. 1F, with a stress factor of 0.166, confirming the reliability of the NMDS analysis results. The significance of differences was tested using three methods, including MRPP (Multiple Response Permutation Procedure), Adonis, and Amova (Analysis of molecular variance). The results showed that both A_MRPP_ and R^2^_Adnois_ were greater than zero, Fs_Amova_ was greater than 1, and all the p-values were less than 0.05(see Table S2), which demonstrated that the difference between groups was greater than that within groups but also emphasized the significance of this difference. We reported the results of Adonis method [nonparametric multivariate analysis of variance (MANOVA)] here: CD vs HC (R2 = 0.015, p = 0.001) and CC vs HC (R2 = 0.006, p = 0.009). In conclusion, the bacterial community composition differed significantly between the healthy and disease samples. Diarrhea associated with a decrease in the diversity of the intestinal microbiota in children while constipation related to an increase.

### *Ruminococcus* was significantly different in abundance between the two disease groups and healthy controls, indicating its potential diagnostic value

**Figure 2:**
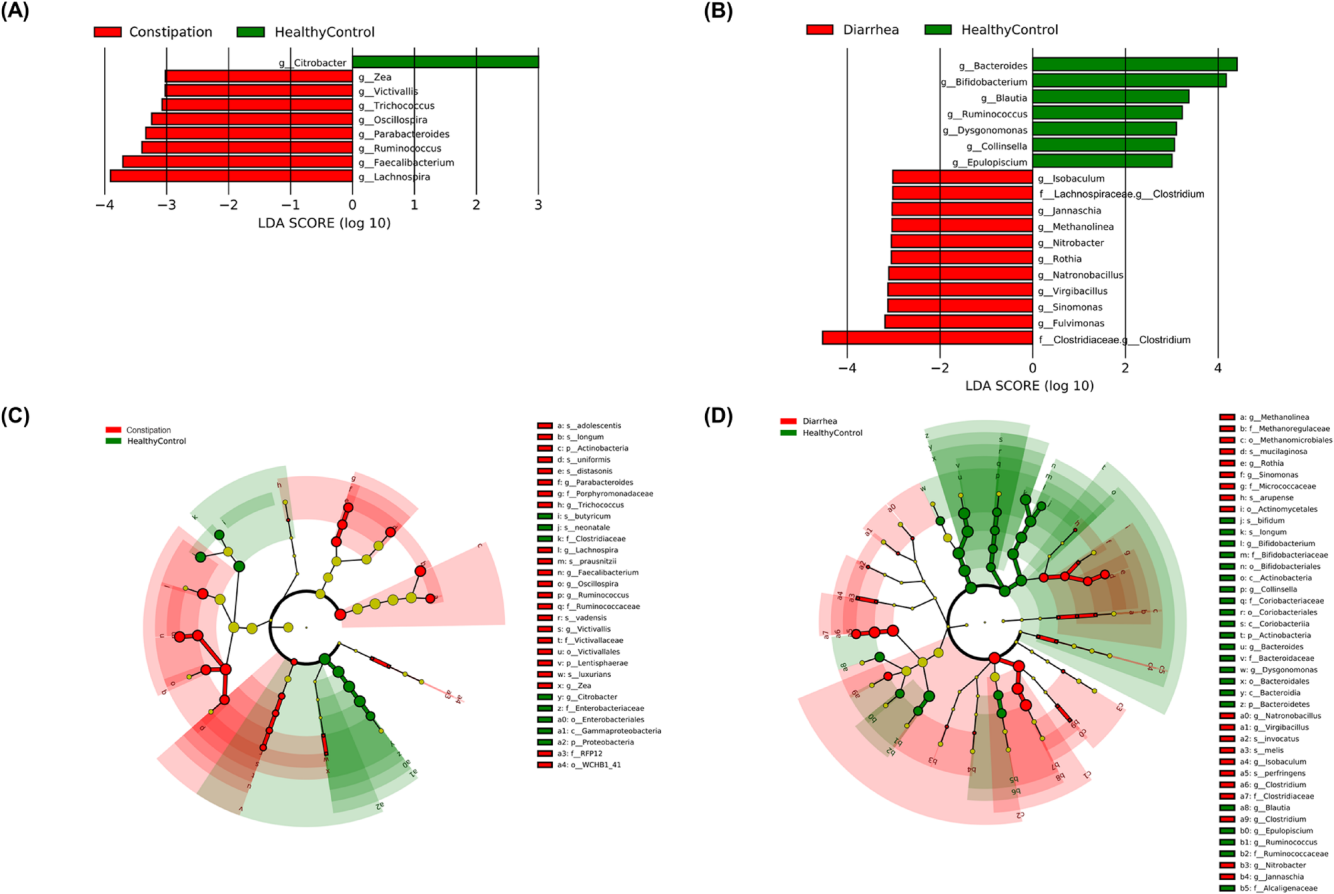
Identification of potential biomarkers using LEfSe. (**A**) LDA score of enriched genera computed by LEfSe analysis between CC and HC groups (LDA score > 3, red, CC; green, HC). (**B**) LDA score of enriched genera computed by LEfSe analysis between CD and HC groups (LDA score > 3, red, CD; green, HC). (**C**) A cladogram made by LEfSe demonstrates different bacterial taxa between the CC and HC groups (red, CC; green, HC). (**D**) LEfSe cladogram of the gut bacterial community obtained from CD and HC groups (red, CD; green, HC).

To further investigate the fecal microbiota in children with constipation and diarrhea, we compared the abundance of different genera with the healthy control. Linear discriminant analysis effect size (LEfSe, version 1.1.2, https://huttenhower.sph.harvard.edu/lefse/)^13^ analysis identified different genus between the CC and HC groups (Fig. 2A), the CD and HC groups (Fig. 2B), with Kruskal–Wallis test (p < 0.05) and linear discriminant analysis (LDA) ≥ 3. Function cladograms are used to claim the structure of bacterial communities in different groups (Fig. 2C, D). The results showed that the genera *Citrobater, Zea, Victivallis, Trichococcus, Oscillospira, Parabacterodies, Ruminococcus*, *Faecalibacterium*, and *Lachnospira* played a major role in distinguishing CC from HC (Fig. 2A), while the genera *Bacteroides, Bifidobacterium, Blautia, Ruminococcus, Dysgonomonas, Collinsella, Epulopiscium, Isobaculum, Lachnospiraceae Clostridium, Jannaschia, Methanolinea, Nitrobacter, Rothia, Natronobacillus, Virgibacillus, Sinomonas, Fulvimonas, and* Clostridiaceae *Clostridium* significantly differed between children with CD and the HC (Fig. 2B). We used MaAsLin2 to examine differential abundance for adjusting the confounding factors, such as gender and age. After correcting for the confounding factors, the differential genera *Citrobacter*, *Oscillospira*, *Parabacteroides*, *Ruminococcus* and *Faecalibacterium* were still observed between the CC and HC group. Additionally, significant differences in the genera *Ruminococcus*, *Collinsella*, *Blautia*, *Rothia*, *Bacteroides*, Clostridiaceae *Clostridium* and *Bifidobacterium* were also founded between the CD and HC group (Table S3). In addition to known differences in genera such as *Bacteroides*^14^, we found that the abundance of *Ruminococcu* significantly increased in the CC group (p < 0.001) and decreased in the CD group at the genus level (p = 0.006). To validate the data consistency between relative and absolute quantification of bacteria, we performed absolute quantification for *Ruminococcus*, which showed a significant increase in the C´C´ group (p < 0.05) and a decrease in the C´D´ group (p < 0.01). In conclusion, our results indicate that both *Ruminococcus* has potential diagnostic value for children with diarrhea and constipation, and the relative balance of *Ruminococcus* in the gut is crucial for maintaining gut health in children.

### *Ruminococcus* may regulate the occurrence of diarrhea and constipation by modulating five metabolic pathways

**Figure 3:**
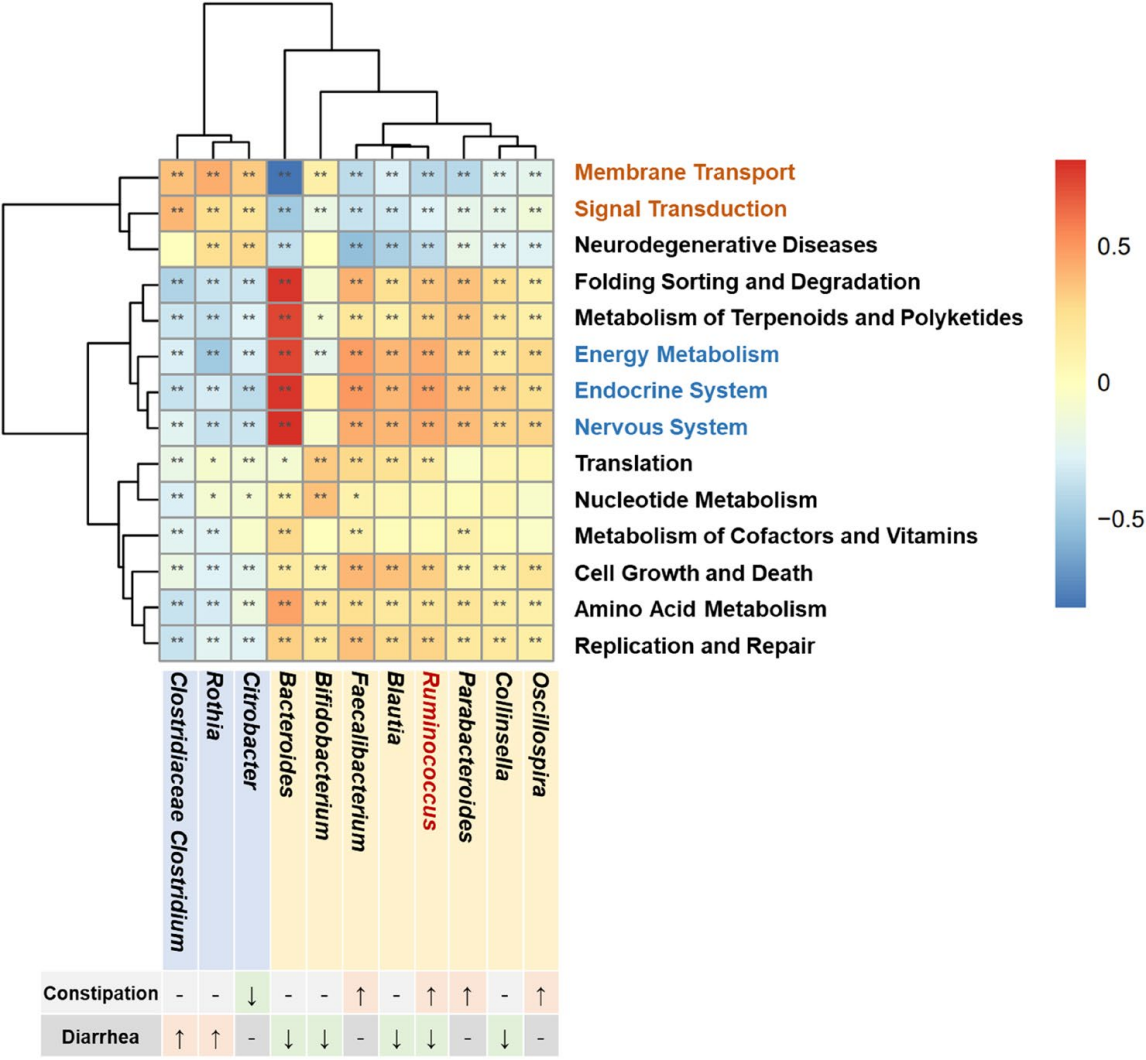
Correlation analysis between significantly different genera and metabolic pathways. A Spearman correlation coefficient analysis was conducted to examine the correlation between the predicted 14 metabolic pathways and the identified genera in the diarrhea and constipation groups. The results are shown in red for positive correlation, blue for negative correlation, and light yellow for no significant correlation.

By performing Picrust analysis and Wilcox rank-sum tests, we identified 14 metabolic pathways distinguishing the CC and HC groups and 29 pathways differentiating the CD and HC groups (see Table S4). Notably, 14 of these metabolic pathways exhibited significant variations in both the diarrhea and constipation groups when compared to the normal controls, as evident from Table 2. We then conducted correlation analyses between these pathways and the bacterial genera that significantly differed between the disease groups and controls using Spearman correlation coefficients (Fig. 3). Here we classified *Oscillospira*, *Parabacteroides*, *Ruminococcus*, and *Faecalibacterium* as high-abundance bacteria with constipation (HABC), while *Citrobacter* was identified as low-abundance bacteria with constipation (LABC). *Rothia* and Clostridiaceae *Clostridium* were categorized as high-abundance with diarrhea (HABD), and *Ruminococcus*, *Collinsella*, *Blautia*, *Bacteroides*, and *Bifidobacterium* as low-abundance with diarrhea (LABD). Three pathways (Membrane Transport, Signal Transduction, Neurodegenerative Disease) showed a positive correlation with HABC and a negative correlation with LABC. Additionally, eight pathways (Folding Sorting and Degradation, Metabolism of Terpenoids and Polyketides, Energy Metabolism, Endocrine System, Nervous System, Cell Growth and Death, Amino Acid Metabolism, Replication and Repair) displayed a positive correlation with LABC and a negative correlation with HABC. Signal Transduction exhibited a positive correlation with HABD and a negative correlation with LABD; while five pathways (Cell Growth and Death, Amino Acid Metabolism, Endocrine System, Nucleotide Metabolism, Replication and Repair) showed a positive correlation with LABD and a negative correlation with HABD. Detailed results of Spearman correlation analysis are listed in Table S5. Specifically, our findings indicated a positive association between *Ruminococcus* and Energy Metabolism, Endocrine System, Nervous System; and a significant negative correlation with Membrane Transport and Neurodegenerative Disease. These results suggest that *Ruminococcus* may affect the occurrence of diarrhea and constipation, two contrasting clinical presentations, by jointly regulating five common pathways including membrane transport, nervous system, energy metabolism, signal transduction and endocrine system pathways, which further supports the potential mechanisms that underlie *Ruminococcus* as diagnostic markers for childhood diarrhea and constipation.

**Table 2:** Fourteen metabolic pathways with significant difference in both the diarrhea and constipation groups as compared to healthy controls.

Pathway	Groups			
	CC vs. HC		CD vs. HC	
	*p	*p_adj	*p	*p_adj
Amino acid metabolism	< 0.001	0.007	< 0.001	< 0.001
Replication and repair	0.001	0.014	< 0.001	< 0.001
Translation	0.001	0.015	< 0.001	< 0.001
Signal transduction	0.002	0.015	< 0.001	< 0.001
Nucleotide metabolism	0.017	0.049	< 0.001	< 0.001
Metabolism of cofactors and vitamins	0.007	0.026	< 0.001	< 0.001
Metabolism of terpenoids and polyketides	0.011	0.035	< 0.001	< 0.001
Energy metabolism	0.001	0.015	< 0.001	< 0.001
Membrane transport	0.005	0.026	< 0.001	< 0.001
Cell growth and death	0.003	0.019	< 0.001	< 0.001
Folding sorting and degradation	0.005	0.026	< 0.001	< 0.001
Endocrine system	0.003	0.035	< 0.001	< 0.001
Nervous system	0.005	0.026	< 0.001	< 0.001
Neurodegenerative diseases	0.011	0.026	0.001	0.001

CC, children with constipation; CD, children with diarrhea.

*Tested via Picrust analysis and Wilcox rank-sum tests.

## Discussion

To investigate the relationship between gut microbiome and childhood diarrhea or constipation, previous studies have focused on microbial biomarkers and structural features within individual diseases. Our study is the first to employ 16S rRNA sequencing to reveal shared features and functional patterns of the gut microbiome in Chinese children aged under three with diarrhea or constipation.

Here we have revealed a significant increase in gut microbiome diversity in children with constipation and a significant reduction in CD compared to HC, consistent with previous studies showing alterations in gut microbiome characteristics in children with diarrhea or constipation^12,15,16^. Diarrhea can lead to gut dysbiosis^17^ damage to the intestinal mucosal barrier^18^, immune system disruption^19^, or malnutrition^20^, all of which may result in a decrease in gut microbiota diversity, and constipation can cause an extended food transit time in the gut, which can alter the gut environment, including pH, redox potential, and the availability of nutrients, thus creating a new microbial habitat. Importantly, the intestinal microbiota plays an essential role in these two diseases with contrasting clinical characters, however, the underlying mechanisms are unclear.

Therefore, we first investigated the microbial α- and β-diversity to determine between-group differences in microbial community structure. Compared to the HC group, the CC group demonstrated a significant increase in the levels of *Faecalibacterium*, *Ruminococcus*, *Parabacteroides*, and *Oscillospira*, along with a decrease in *Citrobacter*. *Faecalibacterium* and *Oscillospira* are known as important butyrate producers in the gut. Butyrate, at physiologically relevant levels, can impact the colon by inhibiting smooth muscle contractions, reducing mucin secretion, and improving water and electrolyte absorption. These effects may contribute to the development of dry and hard stools, potentially worsening constipation^21^. Furthermore, *Parabacteroides*, a possible probiotic commonly found in healthy microbial communities^22^, showed an increased presence in the CC group. Interestingly, Zheng et al.^23^ reported the same increase and found a strong connection between *Parabacteroides* and constipation in the 24–55 age group. *Citrobacter*, an opportunistic enterobacter commonly found in the human gastrointestinal tract^24^, was firstly found to have lower abundance in CC. This finding contradicts a previous study by the American Gut Project^25^, which identified *Citrobacter* as the dominant bacterium in constipated patients, although it did not focus on children. However, in mouse models, *Citrobacter* has been shown to disrupt intestinal mucosa, leading to inflammation and diarrhea^26,27^.

In children with diarrhea, significant shifts in gut flora were observed, including increases in Clostridiaceae *Clostridium* and *Rothia*, and decreases in *Bacteroides*, *Bifidobacterium*, *Blautia*, *Ruminococcus*, and *Collinsella*. One of bacterium in HABD, *Clostridium*, is a genus with fewer than 200 species^28^. Our analysis identified a species-level rise in *Clostridium perfringens*, a food-borne pathogen that produces enterotoxins that cause symptoms such as diarrhea and abdominal pain. This bacterium contributes to sporadic diarrhea in children under five years and antibiotic-associated diarrhea (AAD)^29^. However, no significant differences in *Clostridium difficile* were found in the CD group, despite it being a known trigger of AAD in children^30^. *Rothia*, another HABD, has been found to have increased abundance in children with complementary food-induced diarrhea^31^. Additionally, we observed significant differences in *Rothia mucilaginosa*, which is thought to be heavily correlated with inflammatory markers in the duodenum^32,33^. Five genera in LABD are associated with *Clostridium difficile*-associated diarrhea, viral diarrhea, Shigella colonization and severe acute malnutrition (SAM)^20^. It is noteworthy that some of the LABD, such as *Bifidobacterium* (including *Bifidobacterium bifidum*, *Bifidobacterium adolescentis*, and *Bifidobacterium longum*) and *Blautia* (specifically *Blautia producta*), were probiotics. Undoubtedly, this discovery will provide important insights into the diagnosis and management of children with diarrhea.

At present, clinical practice guidelines and numerous studies recommend that probiotics may be used as adjuncts to effectively alleviate and treat diarrhea^34–36^. For instance, *Bifidobacterium* supplementation can lead to faster normalization of gut flora in children with diarrhea and reduce hospitalization duration for acute diarrhea^37^. Certain probiotics, like *Bifidobacterium longum* JDM301, have been shown to reduce the amount and toxin of *Clostridium difficile* in the cecum^38^. *Bifidobacterium bifidum* FSDJN7O5 and *Bifidobacterium breve* FHNFQ23M3 could relieve the symptoms of diarrhea from *Enterotoxigenic Escherichia coli* (ETEC) infection by reducing the stool water content, restoring the villi structure in the jejunum and ameliorating the fecal short-chain fatty acid (SCFA) content^39^. Probiotic preparations have the potential to modify the gut microbiome and promote normal physiology, ultimately helping to alleviate constipation. Linlin Wang et al. found that a combination of multiple strains of *Bifidobacterium* with adhesion properties (CMB1) effectively improved constipation by enhancing water, propionate, and butyrate levels in feces, as well as overall gastrointestinal transit time^40^. Similarly, Makizaki et al.^41^ discovered that *Bifidobacterium bifidum G9-1 (BBG9-1)* could improve the counts, weights, and water contents of fecal, and alleviate dysbiosis and constipation, suggesting its potential application in constipation caused by a low-fiber diet.

Intriguing, our study specially identified that a significant reduction in *Ruminococcus* levels in children with diarrhea and an increase in children with constipation. *Ruminococcus* is a group of gram-positive bacteria that plays a crucial role in fiber degradation and SCFA synthesis in the gut, reflecting changes in gut ecology and participating in regulating gut health. This contrasting trend of *Ruminococcus* abundances between two groups of children with different diseases suggests that *Ruminococcus* have the potential to assist in diagnosis of pediatric diarrhea and constipation, and that maintaining their levels in a relatively stable range may help to promote gut health. Additionally, pathway analysis suggests *Ruminococcus* may regulate the occurrence of diarrhea and constipation through certain common metabolic pathways, reflecting the complexity of mechanisms in pediatric diarrhea and constipation.

Here we delve into the functional abnormalities observed in membrane transport, nervous system, endocrine system, energy metabolism, and signal transduction in childhood diarrhea and constipation. (1) Membrane transport. The balance of intestinal electrolyte transport, governed by ion secretion and uptake processes, hinges entirely on the basolateral Na/K ATPase^42^. Disruption of this equilibrium can manifest in either diarrhea or constipation. (2) Nervous system. The burgeoning recognition of the ‘gut-brain axis’ underscores the pivotal role of alterations in gastrointestinal microbiota composition as a trigger for various neurocognitive disorders, including autism spectrum disorder (ASD) in children. Notably, children with ASD exhibit constipation at a rate four times higher than their non-ASD counterparts^37^. (3) Endocrine system. Seminal research highlighted the collaborative regulation of gastrointestinal functions by enteroendocrine cells (EECs) and the enteric nervous system. EECs detect luminal and circulating nutrients and subsequently release hormones to modulate satiety, digestion, and glucose homeostasis. Meanwhile, EECs interact with enteric neurons, endothelial cells, and the gastrointestinal epithelium to enhance the digestion and absorption of nutrients. Depletion of EECs culminates in chronic malabsorptive diarrhea, underscoring the central role of EECs in governing nutrient absorption in the gut^43^. (4) Energy metabolism. While there is limited direct evidence regarding the relationship between energy metabolism and constipation or diarrhea in children, a review of clinical studies on the effectiveness of probiotics in Parkinson's disease revealed that probiotics can enhance glucose metabolism (resulting in reduced insulin resistance), decrease peripheral inflammation (leading to lower levels of peripheral TNF-α expression and C-reactive protein), and improve both motor and non-motor functions (resulting in a reduction in overall Parkinson’s disease symptoms and constipation)^44^. (5) Signal transduction. For instance, rotavirus infection and its enterotoxin NSP4 trigger enterochromaffin cells to release serotonin (5-HT), which then acts on the serotonin receptor 5-HT3. This receptor plays a key role in nerve signaling that regulates gut motility, intestinal secretion, and communicates with the brain through the vagus nerve. Additionally, the 5-HT3 receptor contributes to rotavirus-induced diarrhea by enhancing bowel motility^45^. A recent study in SCIENCE revealed that rotavirus utilizes paracrine purinergic signaling to induce intercellular calcium waves (ICWs), which further amplified the dysregulation of host cells and altered gastrointestinal physiology to cause diarrhea^46^. Moreover, SCFAs, the most abundant and critical metabolites of the intestinal microbiota, act as second messengers to promote signal transduction and influence the development of various diseases such as intestinal injuries^47^.

There are some limitations here as we solely focus on the fecal microbiome as a representation and highlights microbial genera, composition, and function in the gut microbiome. Therefore, further exploration and investigation of the metabolome and transcriptome aspects are necessary. Additionally, we have found that *Ruminococcus* can impact the occurrence of diarrhea and constipation through regulating several metabolic system pathways, but further in vitro experiments are needed to validate these findings. Future work should aim to discover the functional mechanisms of the gut microbiome to better understand the relationship with gut health. In certain aspects, such as gathering clinical data, we did not thoroughly investigate the etiology of the child's illness, specifically whether the child with diarrhea had a pathogenic infection or was affected by factors like weaning or diet. As a result, we were unable to accurately categorize the population. Furthermore, our data analysis did not utilize the latest version of software, potentially impacting the accuracy of our analysis results. To address the limitations in analysis, we conducted quality control and denoising of all 16S rRNA data from the samples using the QIIME2 pipeline^48^ (version qiime2-2022.8; https://qiime2.org/). Species annotation was carried out utilizing the Greengenes 22.10 database. The results of these analyses can be seen in File S1. On the one hand, QIIME2 (version qiime2-2022.8; https://qiime2.org/), equipped with the DADA2 denoising method, was utilized for rigorous quality control and identification of amplicon sequence variants (ASVs) stemming from amplified variable regions, in contrast to QIIME1 pipeline (version 1.91; http://qiime.org/). With a strict quality control standard, 31 samples (including 9 CC, 6 CD and 16 HC) were excluded due to a low read count. On the other hand, the Greengenes database has undergone a significant upgrade from version 13.8 to 22.10, which necessarily introduces certain discrepancies in the results. Consequently, the composition of the microbiome may undergo changes based on updated versions of the database and pipeline tools. However, both methods yielded consistent results, showing significantly higher bacterial alpha diversity in CC and lower diversity in CD. Beta diversity also revealed a shift in bacterial communities among different groups experiencing constipation or diarrhea. Specifically, children with constipation displayed higher abundances of *Parabacteroides*, *Ruminococcus*, and *Faecalibacterium,* while *Citrobacter* was less abundant. The absence of two probiotics, *Blautia* and *Bifidobacterium*, was observed in children with diarrhea. Interestingly, a decrease in the abundance of *Bifidobacterium* was noted in the CD group, while an increase was observed in the CC group, underscoring the diagnostic value and efficacy of probiotics in childhood diarrhea. However, the elevated abundance of *Bifidobacterium* was not deemed significant by the QIIME1 pipeline (version 1.91; http://qiime.org/). Additionally, an increased abundance of *Ruminococcus* was identified in constipation, but a non-significant decrease was observed in diarrhea by the QIIME2 pipeline (version qiime2-2022.8; https://qiime2.org/). Nonetheless, the difference in *Ruminococcus* levels between children with constipation and diarrhea was validated in a separate validation cohort population. Despite the limitations of this study, we expect that the findings on changes in the gut flora of children with diarrhea and constipation will be valuable in informing their clinical treatment.

## Methods

### Study population

We enrolled 66 children with diarrhea, 138 children with constipation and 414 healthy children (age < 3 years) from June 2017 to September 2021 at Beijing Dr. Cui Yutao Children's Health Management Center. Children were enrolled if they met the inclusion criteria and did not meet exclusion criteria. Exclusion criteria for children with CC group include (1) male and female (aged 1–3 years old); (2) meet at least 2 of the following symptoms, lasting at least 1 month: (1) the number of defecations is less than or equal to 2 times per week; (2) history of massive fecal retention; (3) history of pain and difficulty in defecation; (4) history of fecal discharge; (5) there are a large number of fecal masses in the rectum; The following additional criteria may be used for children who have learned to defecate: (6) stool incontinence at least once a week after learning to defecate; (7) there is a history of fecal discharge, and it may even cause toilet blockage. And the exclusion criteria for children with CD group included (1) consistent with the diagnosis of diarrhea; (2) male and female (aged 1–3 years old); (3) Exclude organic diseases, meet the Rome IV standards, and defecate more than or equal to three times a day; (4) According to the Bristol fecal Trait Scale, type 6 and type 7 can be regarded as diarrhea; (5) No drugs affecting gastrointestinal motility have been used in the past week. In addition, children were excluded if they (1) had metabolic or neuropsychiatric conditions, cancer, congenital heart disease, liver and kidney dysfunction, or other severe diseases; (2) were taking antibiotics, probiotics, prebiotics, nonsteroidal anti-inflammatory drugs (NSAIDs), opioids, traditional Chinese medicine (TCM), proton pump inhibitors (PPIs), or histamine receptor antagonists during the preceding month before sample collection; and (3) were on a restricted diet, including low-fat diet and vegetarian diet. Another independent population comprised 390 healthy children, 191 children with constipation, and 73 children with diarrhea which were recruited as the same criteria for qPCR validation. This study was performed with the approval of the Ethical Committees of Beijing Institute of Microbiology and Epidemiology (Ethics number: AF/SC-08/02.301), and written informed consent from the guardians of all the participants was obtained. All methods were performed in accordance with the relevant guidelines and regulations.

### Sample collection and DNA extraction

Sterile fecal sampling tubes (SARSTEDT AG & Co. KG, Nümbrecht, Germany) were used to collect approximately 5 mL of feces per participants, minimizing the risk of bias. Fecal samples were frozen immediately after collection and stored at − 80 °C. The samples genomic DNA was extracted using the TIANamp Stool DNA Kit (Tiangen Angen Biotech (Beijing) Co., Ltd., Beijing, China) according to the manufacturer’s instructions. Before subjecting to sequencing, we assessed the quality and purity of DNA using a Qubit R3.0 Fluorometer (Thermo Fisher Scientific Inc., Waltham, Massachusetts, USA), with pure DNA having an OD260/OD280 ratio between 1.8 and 2.0 and all DNA concentrations being higher than 2.5 ng/μL.

### 16S rRNA gene sequencing

All the recruited 618 participants underwent 16S rRNA sequencing, and one-step PCR was used to prepare the PCR Illumina sequencing libraries with the forward and reverse primers for the V3–V4 region (333 nmol each) and KAPA Hi-Fi PCR master mix (Kapa Biosystems, Boston, MA, USA). The forward and reverse primers used were 5′-CCTAYGGGRBGCASCAG-3′ and 5′-GGACTACNNGGGTATCTAAT-3′, respectively. The PCR conditions included an enzyme activation step at 95 °C for 3 min, followed by 20 cycles of 15 s at 98 °C, 30 s at 50 °C, 40 s at 72 °C, and 10 min at 72 °C, with a final hold at 10 °C. The cDNA was purified using Clean Beads (Beckman Coulter Inc., Brea, California, USA) and sequenced on an Illumina HiSeq2500 platform (Illumina, Inc., San Diego, California, USA) to generate approximately 4.5 million reads of 16S rRNA V3–V4 amplicons, including the partial C3 region (341F, 17 base pair (bp)), full V3 region (57 bp), full V4 region (62 bp), and partial C5 region (806R, 20 bp).

### Raw data filtering, classification, and annotation

Adaptors and PCR primers were eliminated from the reads, and paired-end reads were merged using FLASH version 1.2.11. Reads were truncated if they had three consecutive base calls with a quality score below 20, and only high-quality reads accounting for over 75% of the input read length (per single-end read) were included for further analysis. Chimeric reads were identified and removed with USEARCH version 7.0. The reads were clustered into OTUs with QIIME software version 1.9.1^49^ (http://qiime.org/) at an identity threshold of 0.97^50^. OTUs with a count below four were excluded from the analysis. OTUs were annotated to their closest taxonomic neighbors using QIIME (version 1.91; http://qiime.org/) based on the Greengenes database version 13.8^51^.

### Analysis of diversity and microbiota differences

To compare the microbial community richness and evenness among different groups, three α-diversity indices (Chao1, Shannon, and Simpson) were utilized. The QIIME (version 1.91; http://qiime.org/) diversity alpha plugin calculated alpha diversity measures for different groups. Beta diversity was computed using unweighted Unifrac distance based on the profiling table^52,53^. MRPP, Adonis, and Amova were used to evaluate inter- and intra-group differences. The software tools of LEfSe (version 1.1.2; https://huttenhower.sph.harvard.edu/lefse/) were used to identify the biomarkers with significant differences in each group. And the default screening value for the LDA score was set to 3, which can be used to compare two or more groups. Picrust^54^ was used to annotate the metabolic pathways of the genera and the Wilcox rank-sum test identified differential metabolic pathways between the disease groups and healthy controls. Additionally, the correlation between genus and pathway was analyzed using Spearman correlation coefficient.

### Quantification of *Ruminococcus* by qPCR

A total of 487 participants underwent qPCR testing to confirm the absolute quantification of *Ruminococcus* levels. The qPCR was conducted using the Direct Detect Seven Genus/Species Gut Microbes Detection Kit (PCR-Fluorescence Probe, Coyote Bioscience Inc., China) with a PCR program including 10 cycles at 50 °C for 5 s, 95 °C for 5 s, followed by 40 cycles at 95 °C for 50 s and 60 °C for 30 s. The amplification was performed on an ABI 7500 Real-Time PCR System (Applied Biosystems, Foster City, USA), and a PCR standard curve was used to absolutely quantify the *Ruminococcus* in the validation groups.

### Supplementary Information

Supplementary Information.

## Data Availability

The raw sequence data have been deposited in the Genome Sequence Archive^55^ in National Genomics Data Center^56^, China National Center for Bioinformation/Beijing Institute of Genomics, and Chinese Academy of Sciences (GSA: CRA010558). It can be accessible at https://ngdc.cncb.ac.cn/gsa/s/mJ362wah.
