# Supplementary material for: Microbial imbalance in Chinese children with diarrhea or constipation
Source: Sci Rep. 2024 Jun 12;14:13516. doi: 10.1038/s41598-024-60683-6 (PMC11169388; doi:10.1038/s41598-024-60683-6)
Supplement: Supplementary file 1 — Supplementary Information. [file 41598_2024_60683_MOESM1_ESM.zip › Fig S3 A cladogram made by LEfSe demonstrates different bacterial taxa between the CC and HC groups.pdf]

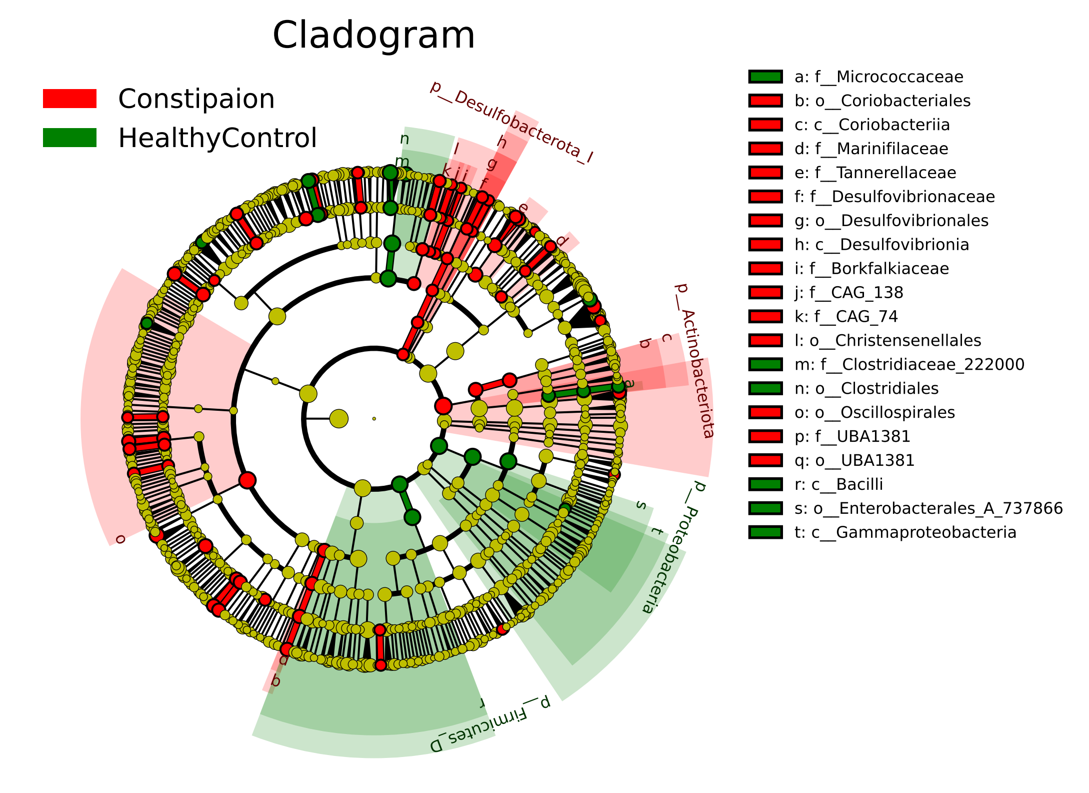
Cladogram
Constipaion
HealthyControl
p__Desulfobacterota_I
p__Actinobacteriota
p__Proteobacteria
p__Firmicutes_D
a: f__Micrococcaceae
b: o__Coriobacteriales
c: c__Coriobacteriia
d: f__Marinifilaceae
e: f__Tannerellaceae
f: f__Desulfovibrionaceae
g: o__Desulfovibrionales
h: c__Desulfovibrionia
i: f__Borkfalkiaceae
j: f__CAG_138
k: f__CAG_74
l: o__Christensenellales
m: f__Clostridiaceae_222000
n: o__Clostridiales
o: o__Oscillospirales
p: f__UBA1381
q: o__UBA1381
r: c__Bacilli
s: o__Enterobacterales_A_737866
t: c__Gammaproteobacteria
