# Supplementary material for: Microbial imbalance in Chinese children with diarrhea or constipation
Source: Sci Rep. 2024 Jun 12;14:13516. doi: 10.1038/s41598-024-60683-6 (PMC11169388; doi:10.1038/s41598-024-60683-6)
Supplement: Supplementary file 1 — Supplementary Information. [file 41598_2024_60683_MOESM1_ESM.zip › Fig S4 A cladogram made by LEfSe demonstrates different bacterial taxa between the CD and HC groups.pdf]

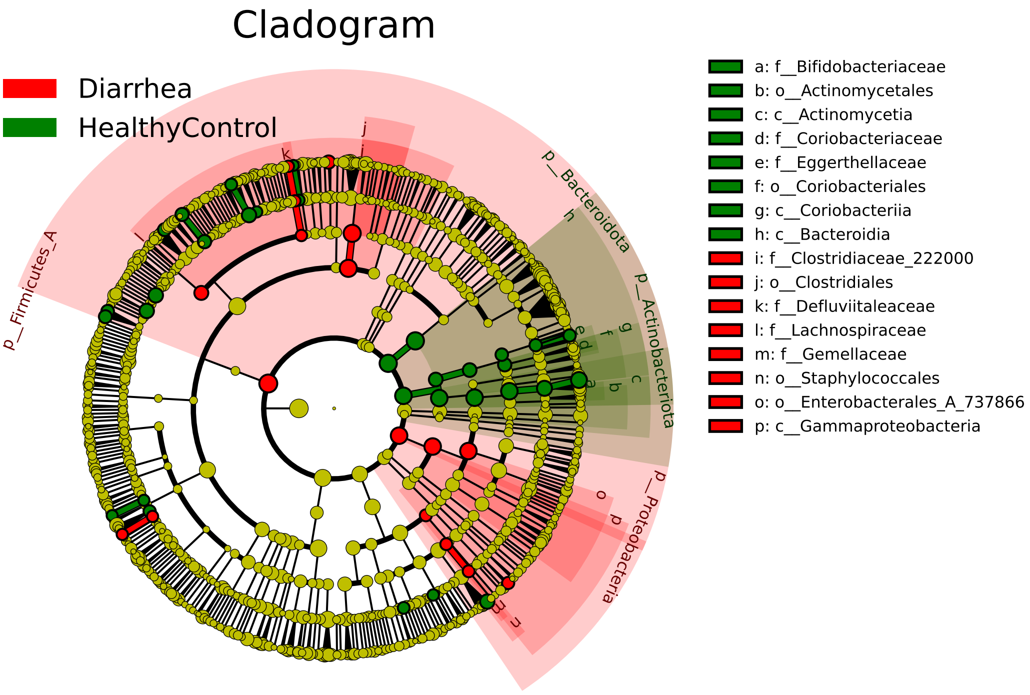
Cladogram
Diarrhea
HealthyControl
a: f__Bifidobacteriaceae
b: o__Actinomycetales
c: c__Actinomycetia
d: f__Coriobacteriaceae
e: f__Eggerthellaceae
f: o__Coriobacteriales
g: c__Coriobacteriia
h: c__Bacteroidia
i: f__Clostridiaceae_222000
j: o__Clostridiales
k: f__Defluviitaleaceae
l: f__Lachnospiraceae
m: f__Gemellaceae
n: o__Staphylococcales
o: o__Enterobacterales_A_737866
p: c__Gammaproteobacteria
p__Firmicutes_A
p__Bacteroidota
p__Actinobacteriota
p__Proteobacteria
