# Supplementary material for: Microbial imbalance in Chinese children with diarrhea or constipation
Source: Sci Rep. 2024 Jun 12;14:13516. doi: 10.1038/s41598-024-60683-6 (PMC11169388; doi:10.1038/s41598-024-60683-6)
Supplement: Supplementary file 1 — Supplementary Information. [file 41598_2024_60683_MOESM1_ESM.zip › File S1 The re-analysis results of CC and CD.docx]

**The** **re-analysis results of** **children constipation or diarrhea**

**(QIIME2 v2022.8 and Greengenes v22.10)**

**1. Methods**

**1.1 Raw data filtering, classification, and annotation**

The adaptors and PCR primers were removed from the reads, and these paired-end reads were denoised, filtered and joined using the DADA2 software package implemented in QIIME 2(version qiime2-2022.8; https://qiime2.org/), and the number of reads from each sample was rarefied to 2000. Rare amplicon sequence variants (ASVs) with total frequency less than 5, or was observed in one sample, or relative abundance was below 0.1% were removed. Taxonomy was assigned to ASV using the feature-classifier, a classify-sklearn naive Bayes taxonomy classifier using machine learning against the Greengene 22.10 database.

**1.2 Diversity analysis and variation analysis**

The QIIME2 diversity alpha plugin produced alpha diversity measures (Chao1 richness index, Shannon and Simpson diversity index), which were used to analyze the alpha diversity level of different groups. The differences of alpha diversity were further tested using Kruskal–Walli’s analysis (K–W test). The unweighted Unifrac distance matrices between samples were used for non-metric multidimensional scaling analysis (NMDS) at the ASV level.

**1.3 Analysis of microbiota differences**

The software tools of LEfSe (version 1.1.2; https://huttenhower.sph.harvard.edu/lefse/) were used to identify the biomarkers with significant differences in each group. And the default screening value for the LDA score was set to 3, which can be used to compare two or more groups.

**2.Results**

**2.1 16S rRNA Diversity Analysis**

A total of 618 samples [Children with constipation (CC, n=138), Children with diarrhea (CD, n=66), Healthy control (HC, n=414)] were analyzed by 16S rRNA sequencing. After joint and quality filtering, a total of 47,581,050 reads passed the filters applied through the DADA2 plugin in the QIIME2 software package, with an average value of 76,992 reads/sample. Following primer trimming and raw read quality filtering, samples from three group were analyzed separately. In order to avoid biases due to different sequencing depths, 31 samples (including 9 CC, 6 CD, and 16 HC) were excluded from further analyses due to a low read count.

The diversity of gut microbiota was significantly different among three groups. Higher chao1, shannon and simpson index were observed in CC group than that in HC group. And Lower chao1, shannon and simpson index were identified in CD group (Table S6, Fig S1). The NMDS analysis based on the unweighted Unifrac distances of microbial communities in the samples was shown in Fig S2.


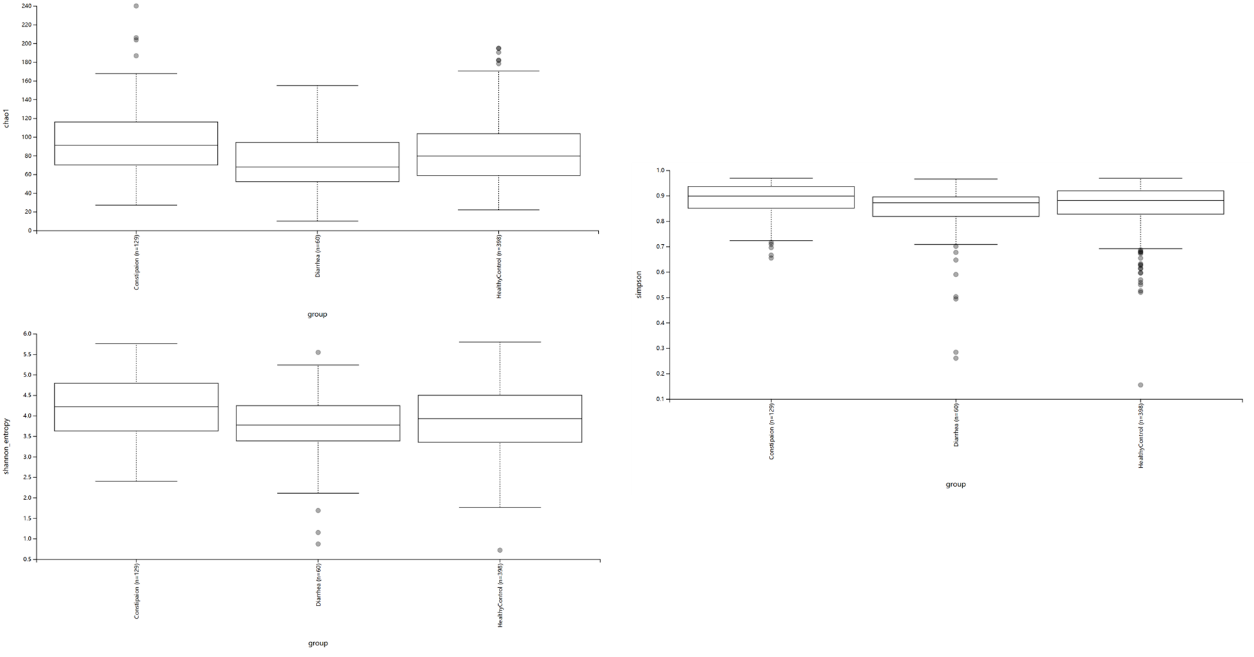


**Fig S1 The alpha diversity re-analysis results among three groups**


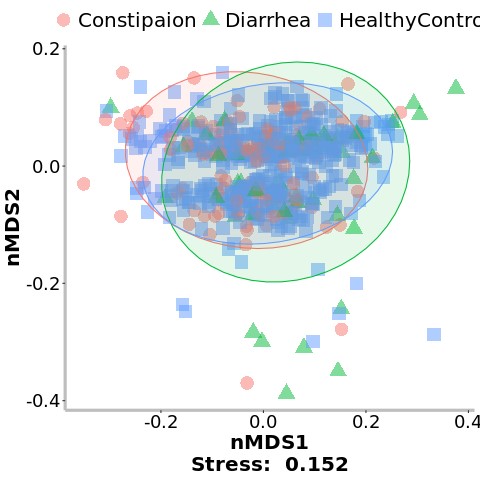


**Fig S2 The re-analysis results of Beta-diversity index among the three groups (plots of NMDS)**

**2.3 Different genera in disease groups**

Function cladograms are used to claim the structure of bacterial communities in different groups (Fig S3, Fig S4). The results showed that the genera *Bacteroide*, *Lawsonibacter*, *Fimenecus*, *Bifidobacterium*, ***Parabacterodies***, ***Ruminococcus***, ***Faecalibacterium***, *Anaerostipes*, *Fusicatenibacter*, *Fimenecus*, ***Clostridium***, *Agathobacter* and ***Rothia*** played a major role in distinguishing CC from HC (Table S7), while the genera ***Bifidobacterium***, ***Blautia***, *Mediterraneibacter*, *Hungatella*, and *Streptococcus* significantly differed between children with CD and the HC (Table S8). The bold genera are consistent with the results of QIIME 1 pipeline (version 1.91; http://qiime.org/).


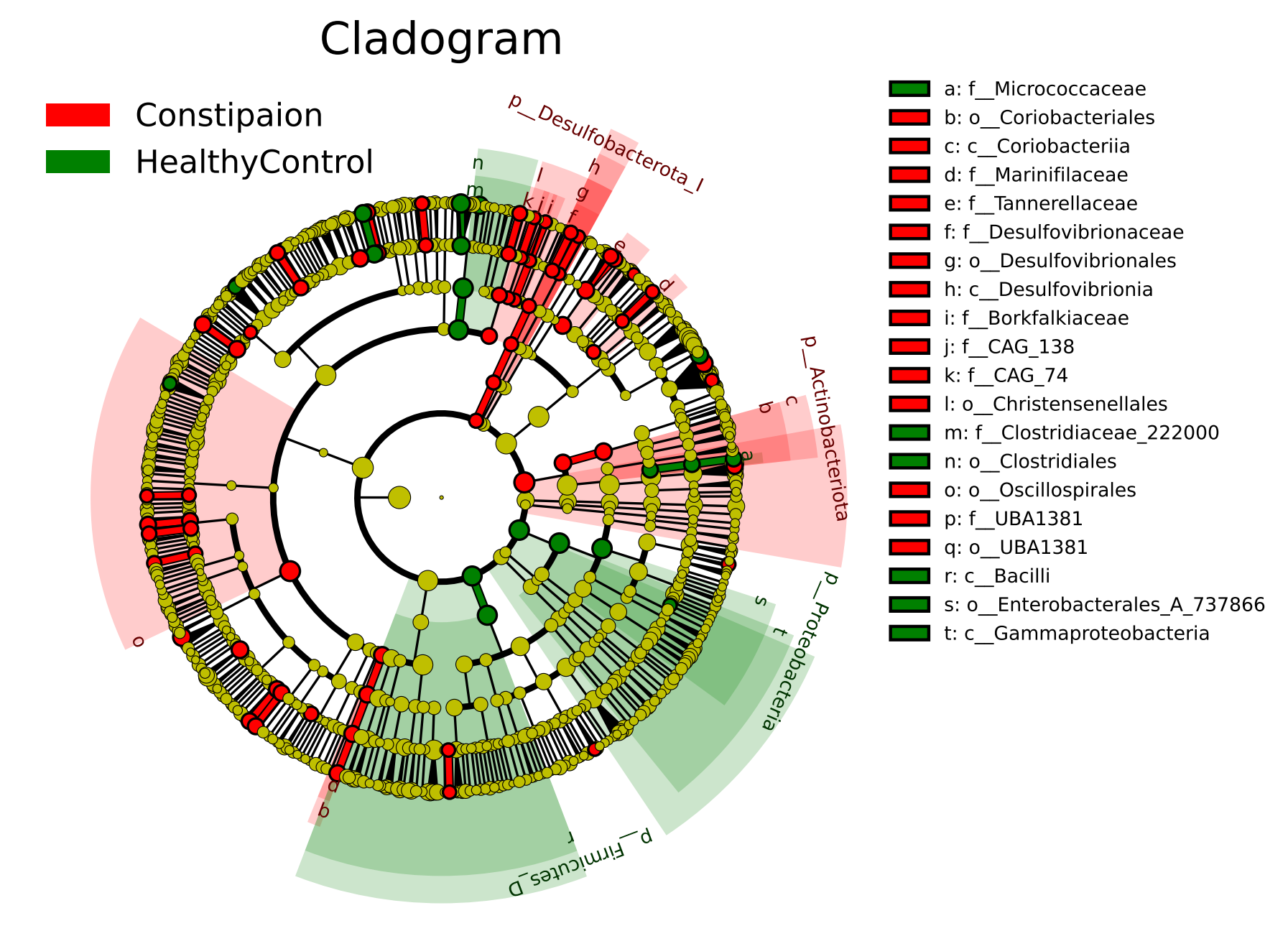


**Fig S3 A cladogram made by LEfSe demonstrates different bacterial taxa between the CC and HC groups (red, CC; green, HC)**


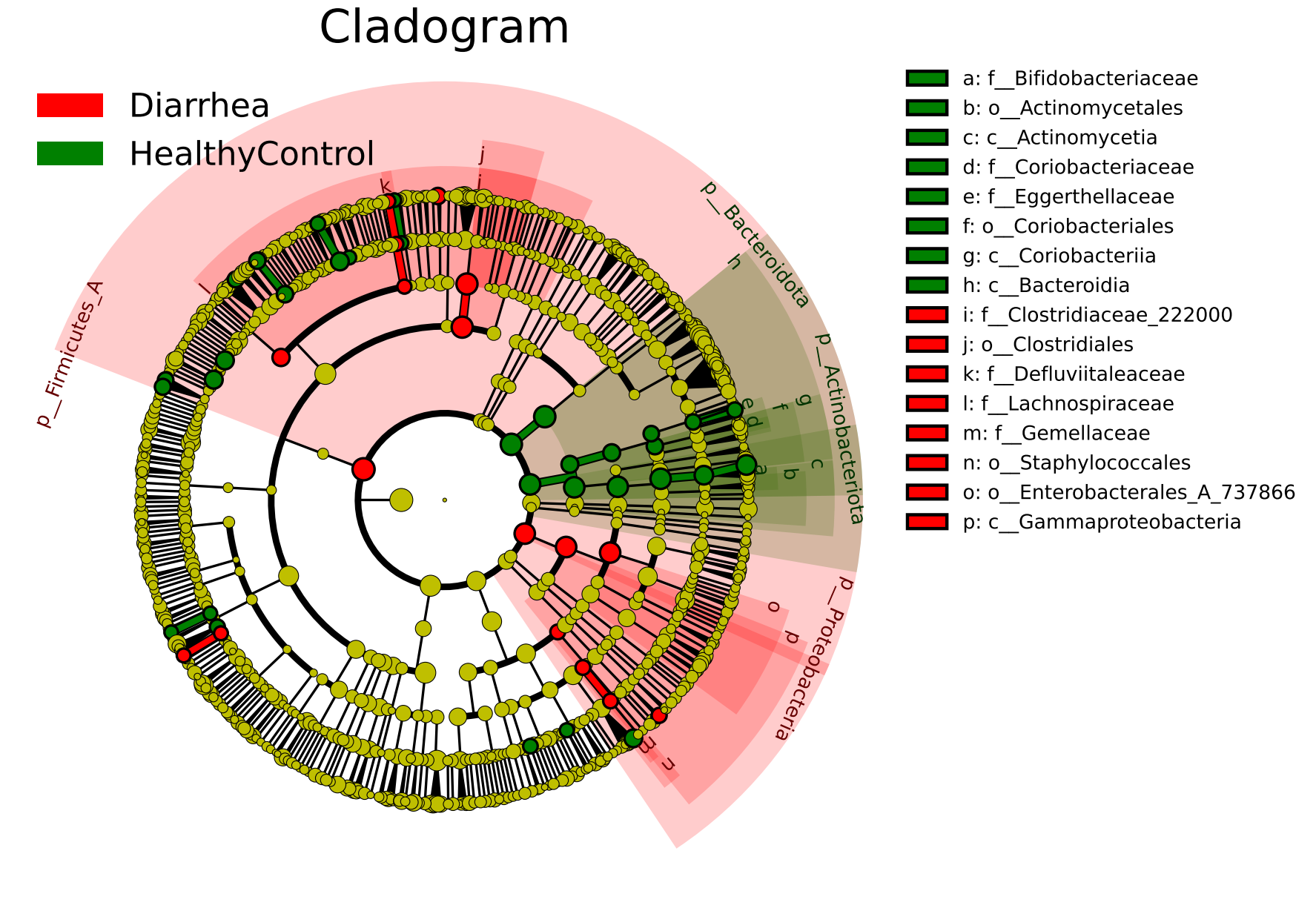


**Fig S4 LEfSe cladogram of the gut bacterial community obtained from CD and HC groups (red, CD; green, HC)**
