# Supplementary material for: Microbial imbalance in Chinese children with diarrhea or constipation
Source: Sci Rep. 2024 Jun 12;14:13516. doi: 10.1038/s41598-024-60683-6 (PMC11169388; doi:10.1038/s41598-024-60683-6)
Supplement: Supplementary file 1 — Supplementary Information. [file 41598_2024_60683_MOESM1_ESM.zip › Table S2 The Significance tests with three different statistical approaches.docx]

**TABLE S2 The Significance tests with three different statistical approaches.**

| **Group** | **MRPP** | | **Adnois** | | **Amova** | |
| --- | --- | --- | --- | --- | --- | --- |
|  | **A** | **P-value** | **R^2^** | **P-value** | **Fs** | **P-value** |
| **CC vs HC** | 0.003 | 0.010 | 0.006 | 0.009 | 3.580 | 0.011* |
| **CD vs HC** | 0.008 | 0.001 | 0.015 | 0.001 | 7.341 | <0.001* |
| **CC vs CD** | 0.035 | 0.001 | 0.064 | 0.001 | 13.775 | <0.001* |

**Table S2** shows that both A_MRPP_ and R^2^_Adnois_ were greater than zero, Fs_Amova_ was greater than 1, and all the P-values were less than 0.05. These results indicated that the difference between groups was greater than that within groups, and such difference was significant (p < 0.05).
