# Supplementary material for: Microbial imbalance in Chinese children with diarrhea or constipation
Source: Sci Rep. 2024 Jun 12;14:13516. doi: 10.1038/s41598-024-60683-6 (PMC11169388; doi:10.1038/s41598-024-60683-6)
Supplement: Supplementary file 1 — Supplementary Information. [file 41598_2024_60683_MOESM1_ESM.zip › Table S6 The alpha diversity re-analysis results .docx]

**Table S6 The alpha diversity re-analysis results**

| **Group** | **Chao1** | | **Shannon** | | **Simpson** | |
| --- | --- | --- | --- | --- | --- | --- |
|  | **H** | **p** | **H** | **p** | **H** | **p** |
| **CC vs HC** | 12.280 | <0.001 | 12.275 | <0.001 | 8.809 | 0.003 |
| **CD vs HC** | 5.292 | 0.021 | 2.789 | 0.095 | 3.061 | 0.080 |
