# Supplementary material for: Microbial imbalance in Chinese children with diarrhea or constipation
Source: Sci Rep. 2024 Jun 12;14:13516. doi: 10.1038/s41598-024-60683-6 (PMC11169388; doi:10.1038/s41598-024-60683-6)
Supplement: Supplementary file 1 — Supplementary Information. [file 41598_2024_60683_MOESM1_ESM.zip › Table S7 The re-analysis results of different genera in CC vs HC.docx]

**Table S7 The re-analysis results of different genera in CC vs HC**

| **Biomarker names** | **LDA** | **p** |
| --- | --- | --- |
| **Constipation** | | |
| f__Bacteroidaceae.g__Bacteroides_H.s__Bacteroides_Hovatus | 3.574 | 0.002 |
| f__Ruminococcaceae.g__Faecalibacterium | 3.125 | 0.008 |
| f__Oscillospiraceae_88309.g__Lawsonibacter.s__ | 3.315 | 0.010 |
| f__Ruminococcaceae.g__Ruminococcus_D.s__Ruminococcus_Dbicirculans | 3.155 | 0.016 |
| f__Acutalibacteraceae.g__Fimenecus.s__Fimenecussp000432435 | 3.136 | 0.011 |
| f__Bifidobacteriaceae.g__Bifidobacterium_388775.s__Bifidobacteriumdentium | 3.053 | 0.045 |
| f__Tannerellaceae.g__Parabacteroides_B_862066 | 3.079 | 0.025 |
| f__Lachnospiraceae.g__Anaerostipes | 3.096 | 0.002 |
| f__Ruminococcaceae.g__Ruminococcus_D | 3.153 | 0.016 |
| f__Lachnospiraceae.g__Fusicatenibacter | 3.000 | 0.037 |
| f__Acutalibacteraceae.g__Fimenecus | 3.166 | 0.011 |
| **Healthy** | | |
| f__Bacteroidaceae.g__Bacteroides_H.s__Bacteroides_Huniformis | 3.167 | 0.036 |
| f__Clostridiaceae_222000.g__Clostridium_T.s__Clostridium_Tneonatale | 4.000 | 0.002 |
| f__Clostridiaceae_222000.g__Clostridium_P.s__Clostridium_Pperfringens | 3.643 | 0.025 |
| f__Clostridiaceae_222000.g__Clostridium_T | 3.610 | 0.048 |
| f__Lachnospiraceae.g__Agathobacter_164117.s__Agathobacterrectalis | 3.121 | 0.035 |
| f__Micrococcaceae.g__Rothia.s__Rothiasp001808955 | 3.022 | 0.002 |
| f__Lachnospiraceae.g__Agathobacter_164117 | 3.124 | 0.035 |
| f__Micrococcaceae.g__Rothia | 3.022 | 0.002 |
