# Supplementary material for: Microbial imbalance in Chinese children with diarrhea or constipation
Source: Sci Rep. 2024 Jun 12;14:13516. doi: 10.1038/s41598-024-60683-6 (PMC11169388; doi:10.1038/s41598-024-60683-6)
Supplement: Supplementary file 1 — Supplementary Information. [file 41598_2024_60683_MOESM1_ESM.zip › Table S8 The re-analysis results of different genera in CD vs HC.docx]

**Table S8 The re-analysis results of different genera in CD vs HC**

| **Biomarker names** | **LDA** | **p** |
| --- | --- | --- |
| **Diarrhea** | | |
| f__.g__.s__Clostridiumpiliforme | 3.098 | 0.043 |
| **Healthy** | | |
| f__Bifidobacteriaceae.g__Bifidobacterium_388775 | 3.918 | 0.000 |
| f__Lachnospiraceae.g__Lachnospira | 3.434 | 0.044 |
| f__Lachnospiraceae.g__Blautia_A_141781 | 3.392 | 0.004 |
| f__Lachnospiraceae.g__Mediterraneibacter_A_155507.s__Mediterraneibacter_A_155507faecis | 3.002 | 0.022 |
| f__Bifidobacteriaceae.g__Bifidobacterium_388775.s__Bifidobacteriumlongum | 3.991 | 0.026 |
| f__Lachnospiraceae.g__Hungatella_A_128155 | 3.434 | 0.037 |
| f__Streptococcaceae.g__Streptococcus.s__Streptococcusvestibularis | 3.402 | 0.010 |
