# Supplementary figures and images for: Microbial imbalance in Chinese children with diarrhea or constipation
Source: Sci Rep. 2024 Jun 12;14:13516. doi: 10.1038/s41598-024-60683-6 (PMC11169388; doi:10.1038/s41598-024-60683-6)

## Slide 1
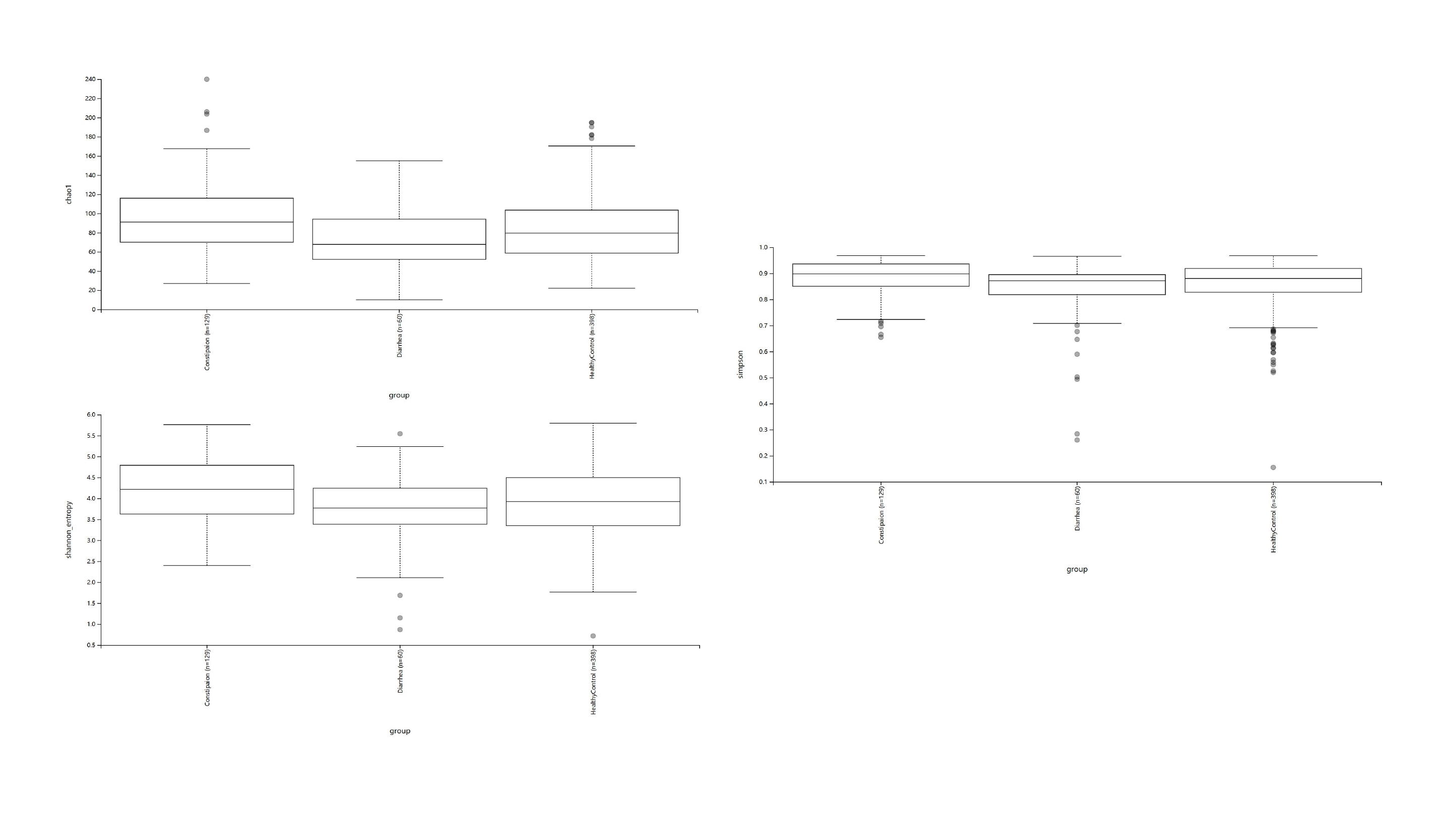

Supplement: Supplementary file 1 — Supplementary Information. [file 41598_2024_60683_MOESM1_ESM.zip › Fig S1 The alpha diversity re-analysis results among three groups.pptx]

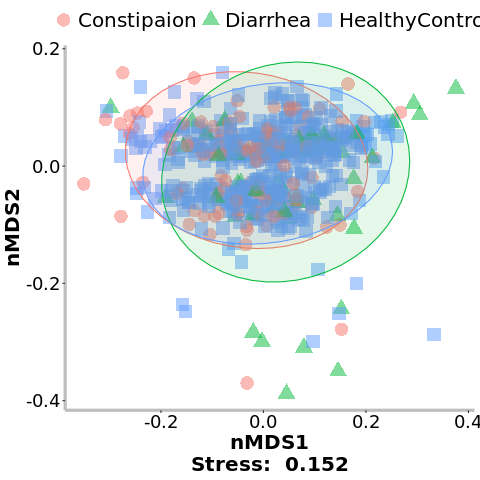

Supplement: Supplementary file 1 — Supplementary Information. [file 41598_2024_60683_MOESM1_ESM.zip › Fig S2 The re-analysis results of Beta-diversity index among the three groups (plots of NMDS).png]
